# Study of the incidence of osteoporosis in patients with Sjögren’s syndrome (pSS) and investigation of activation of the RANKL /RANK and osteoprotegerin (OPG) system

**DOI:** 10.31138/mjr.29.4.224

**Published:** 2018-12-18

**Authors:** Charalampos Skarlis, Eleni Palli, Andrianos Nezos, Michail Koutsilieris, Clio P. Mavragani

**Affiliations:** 1 Department of Physiology,; 2 Department of Pathophysiology, School of Medicine, National University of Athens, Athens, Greece

**Keywords:** RANKL, RANK, osteoprotegerin (OPG), primary Sjögren’s syndrome, autoimmunity, autoimmune diseases, osteoporosis

## INTRODUCTION

Primary Sjögren’s syndrome (pSS) is a common chronic autoimmune disease affecting 0.5–4.8% of the population. It is characterized by lymphocytic infiltration of exocrine glands - mainly salivary and lacrimal - resulting in oral and ocular dryness, although any organ system can virtually be affected. Among all autoimmune diseases, pSS has the highest risk for development of non-Hodgkin’s lymphoma (NHL) and approximately 10% of patients with SS associated with increased risk for B-cell lymphoma development and high mortality rates. In pSS there are various systemic manifestations; such as arthritis, rashes, Raynaud’s phenomenon, peripheral neuropathy and glomerulonephritis.^1,2^ While over the past few years, the importance of coexistence in the context of systemic autoimmune diseases such as subclinical atherosclerosis and osteoporosis has been extensively studied in pSS, data on the incidence and underlying pathophysiological mechanisms of osteopenia/osteoporosis are limited.

In particular, osteopenia and osteoporosis levels appear to be elevated in patients with systemic autoimmune disorders, such as systemic lupus erythematosus (SLE), rheumatoid arthritis (RA) and systemic sclerosis.^3–5^ Patients with pSS display a set of clinical and serological features that could possibly lead to reduced bone remodelling and reduced bone mineral density (BMD); such as low vitamin D levels, hypercalciuria associated with underlying interstitial nephritis, steroid use, as well as coexistence with other autoimmune disorders associated with an increased risk of osteoporosis such as primary biliary cirrhosis, celiac disease and distal renal tubular acidosis (dRTA).

A strong relationship between the Wingless-type signalling pathway (Wnt) and pathophysiology of osteoporosis has been recognized, as mutations in that pathway lead to reduced bone density.(6) 77 with rheumatoid arthritis (RA A recent study found reduced bone density in pSS patients compared to reduced levels of DKK1 (Dickkopf-related protein 1) in the Wnt pathway, where it may be due to inhibition of bone formation.^7^ Another equally important signalling pathway crucial for bone homeostasis is the molecular pathway of RANKL and osteoprotegerin (OPG).^7,8^ Nf-κB receptor activating factor (RANK), its ligand (RANKL) and osteoprotegerin (OPG) - all members of the cytokine tumour necrosis factor (TNF) superfamily - play an important role in bone homeostasis as principal osteoclastogenic factors.^8^ RANKL is expressed by syno-vial cells, bone marrow stromal cells and osteocytes, and is secreted by osteoblasts and immune cells; such as activated T cells, including Tregs and activated B cells and lymphoid tissue cells.^(9–13)^ The expression of RANKL is modulated by various cytokines upon stimulation by memory B cells, including interleukin-1 (IL-1), IL-6, IL-11, IL-12, IL-15 and TNF-α, glucocorticoids and parathyroid hormone.^12–16^ Osteoprotegerin acts as a competitive antagonist receptor, which binds membrane protein RANK and soluble RANKL molecule, plays an osteoprotective role.^17^ RANKL prevents bone reconstruction while OPG inhibits this action, effectively promoting bone reconstruction. The RANKL / OPG ratio appears to be the key to maintaining bone homeostasis. The RANKL / RANK / OPG signalling pathway has been shown to be activated in many autoimmune disorders, such as RA and systemic lupus erythematosus (SLE), with a role in the development of bone erosions and osteoporosis.^14,18,19^

## AIM OF THE STUDY

Although the link between autoimmune diseases and comorbidities such as osteoporosis is well characterized, the association between Sjögren’s syndrome and osteoporosis has not been fully illustrated. Sjögren’s syndrome is an ideal model of studying the mechanisms of osteoporosis in the context of systemic autoimmunity, since steroid use is not widespread in these patients.

The aim of this study is to investigate the prevalence of osteoporosis in patients with Sjögren’s syndrome and to investigate the possible contribution of the RANKL / RANK / OPG pathway through a comparative study in sicca and healthy controls.

Elucidation of the role of RANK/RANKL/OPG pathway in a subset of these patients would identify a potential target subgroup in which blockade of the pathway using the commercially available agent denosumab would be beneficial.

## MATERIALS AND METHODS

For our study, tissue samples from minor salivary gland biopsy, peripheral blood and serum from SS patients and controls are required. All subjects are followed up at the Rheumatology Unit of the Medical School of the University of Athens, after a briefing and written consent to participate in the study. A collection of at least 80 salivary gland biopsies derived from patients with SS with or without B-cell lymphoma is needed, as well as controls with dry mouth symptoms in which the histopathological criteria for the disease (dry mouth) are not met. All SS samples fulfil the revised international criteria for 2002 for the classification of Sjögren’s syndrome,^20^ with or without co-diagnosis of WHO lymphoma. Age/gender matched healthy controls will also be included. The samples will be collected in the archives of the Physiology Laboratory of the Athens School of Medicine and stored in −80° C. For all patients, Bone Mineral Density (BMD) measurements will be performed by measuring dual energy X-ray bone resorption using the QDR4500 densitometer (Hologic, Bedford, MA, USA) on the hip (total hip region) and the spine (anteroposterior lumbar spine, lumbar vertebrae L1 to L4). The presence of osteoporosis or osteopenia will be defined according to the World Health Organization (WHO) classification system, as a T-score < −2.5 SD and −1 SD, respectively, at either the lumbar spine or the hip. In addition, the complete history of the patients will be recorded including demographics, age of menopause, the number of previous fractures, and the total steroid dose administered; as well as calcium and phosphorus levels in the blood and 24h urine, as well as 25-dihydroxy vitamin D3 and parathyroid hormone (PTH) serum levels.

### RNA extraction

Total RNA will be isolated from minor salivary glands biopsies (MSG), with Qiagen RNeasy mini kit (Qiagen, Chatsworth, CA) or Trizol Reagent (Ambion, Life Sciences, USA) according to the manufacturers’ instructions. All samples will be treated with DNAse I (Qiagen, Germany) prior to cDNA synthesis. The quantity and quality of RNA samples were spectrophotometrically tested (Bio-spec Nano, Japan).

### Reverse Transcription reaction (RT)

0.5μg of total RNA obtained from MSG samples and 0.25μg of total RNA from PAXgene samples will be reverse-transcribed using the Superscript III reverse transcriptase system from Invitrogen (Carlsbad, CA). Oligo-dT primer (0.5μM) (Qiagen, Germany) will be used to amplify mRNA and an RNAse inhibitor will be included to prevent degradation (RNAseOUT, Invitrogen, USA). cDNA samples will be diluted 1:10 and 1:5, respectively with nuclease free water (Qiagen, Germany) immediately after synthesis and stored at −20°C.

### Quantitative Real-Time Polymerase Chain Reaction (q-RT-PCR)

Quantitative Real-Time Polymerase Chain Reaction (qRT-PCR) will be used to quantify specific cDNAs using the Bio-Rad IQ5 thermocycler and the IQ Bio-Rad SYBR Green Supermix (Bio-Rad, Hercules, CA). For the reaction is necessary a total volume of 25 *μ*L per reaction and the reaction mixture included 2 *μ*L of template cDNA of each primer for both RANKL (Forward: TCAGCCTTTTGCTCATCTCACTAT and Reverse: CCACCCCCGATCATGGT) and OPG (Forward: GCTCACAAGAACAGATTTCCAG and Reverse: CTGTTTTCACAGAGGTCAATATCTT).

To assess product specificity, amplicons will be checked by melting curve analysis. Then, the threshold values will be recorded for each sample in the logarithmic portion of the amplification curve. In addition, a reference sample will be included in each PCR plate to ensure normalization across experiments.

### Immunohistochemistry (IHC)

Immunohistochemical detection of RANKL and OPG antigens will be performed in paraffin-embedded tissues using the specific monoclonal antibodies anti-RANKL and anti-OPG (OriGene Scientific, USA) Briefly, paraffin sections will be rehydrated in successive baths of xylene, 100%, 96%, 80%, 70% ethanol, and water. The sections will be washed with PBS (phosphate buffered saline) 3 times. Antigen retrieval will be performed by microwaving for 10 minutes in 0.01 M Citrate buffer (pH 6.0). Incubation for 10 minutes at room temperature with Power Block Universal Blocking Reagent (BioGenex, USA) and 10 minutes with 3% H2O2 (BioGenex, USA) will be performed to block non-specific antibody binding and endogenous peroxidase activity, respectively. Incubation of serial sections with monoclonal anti-mouse anti-RANKL (dilution 1:50 OriGene Scientific USA) and monoclonal anti-OPG (dilution 1:50, OriGene Scientific USA), Reagent (BioGenex, USA) will be performed for 30 minutes at room temperature. Polymer–horseradish peroxidise (HRP) Reagent (BioGenex, USA) will be applied for 30 minutes at room temperature, and, after a washing step, the substrate diaminobenzidine (DAB) solution (BioGenex, USA) will be applied for 5-10 minutes. Biopsy sections will be counterstained with haematoxylin for 2 minutes (Mayers Haematoxylin solution, Sigma Aldrich Inc, USA), dehydrated in successive baths of water, 70%, 80%, 96%, 100% ethanol, and xylene and cover-slip mounted with 2 drops of Aqueous Mounting Media (BioGenex, USA). Negative control staining will be performed by replacing primary antibody with PBS. Positive immunoreactivity appears as brown colour.

### Enzyme-linked immunosorbent assay (ELISA assay)

RANKL and OPG serum levels will be assessed by ELISA, according to manufacturer’s instructions in SS patients and healthy controls.

### Statistical Analysis

Statistical analysis was performed by SPSS v.21 package. Two-group comparisons of continuous data were assessed using t-tests, or the Mann-Whitney test, when the data did not have a normal distribution. Comparisons between groups were performed by Fisher’s exact two tailed test and Mann Whitney test. Difference was considered statistically significant if p<0.05.
